# Safe Disposal of Unused Medicine among Health Professions Students at Makerere University: Knowledge, Practices and Barrier

**DOI:** 10.21203/rs.3.rs-2525937/v1

**Published:** 2023-01-31

**Authors:** Rachel Nakiganda, Fredrick Katende, Ferguson Natukunda, Gloria Joy Asio, William Ojinga, Allan Bakesiga, Claire Namuwaya, Lourita Nakyagaba, Blaise Kiyimba

**Affiliations:** Makerere University; Makerere University; Makerere University; Makerere University; Makerere University; Makerere University; Makerere University; Makerere University; Makerere University

**Keywords:** Unused, Medicines, Disposal, Students, Health, Makerere, University

## Abstract

**Background:**

Environmental contamination with antimicrobial agents is one of the leading drivers of antimicrobial resistance (AMR) worldwide. However, scarce data exists concerning the factors fueling unsafe disposal of medicines globally. This study aimed to assess for the knowledge, practices, and barriers concerning safe disposal of unwanted medicines among health professions students (HPS) at Makerere University, Uganda.

**Methods:**

We conducted a cross-sectional study using an online assessment tool sent through WhatsApp groups and E-mail addresses of undergraduate HPS at the College of Health Sciences Makerere University, Uganda between 1^st^February and 16^th^March 2022.Good knowledge was defined as a score of 80% of the knowledge domain questions on a standardized questionnaire.

**Results:**

We enrolled 205 participants, 135 (65.9%) were male, with a median age of 23 (range: 19 –43) years. Overall, 157 (76%) participants had good Knowledge. The mean knowledge score was 63%. About half (n=102, 49.8%) of the participants had unused medicines kept at their respective residential places, mainly antibiotics (27%, ×/102) and analgesics (21 %, ×/102). The most common method of medicine disposal was dumping into household garbage (n=103, 50.2%). Only 37 (18%) of participants had ever used the take-back method of medicine disposal. The most reported barriers for safe disposal were inadequate knowledge and insufficient advice from the dispensers concerning safe disposal practices. Year of study was the only factor significantly associated with knowledge about safe disposal of medicines, (adjusted Odds Ratio: 9.7, 95%CI 2.3 – 40.5, p= 0.002), with participants in higher academic years having more knowledge than those in lower years.

**Conclusion:**

Despite a good knowledge level among the participants, the practice of safe disposal of unused medicines remains suboptimal in this population. Strategies aimed at enhancing safe disposal practices such as giving proper instructions to medicine buyers by dispensers regarding disposal practices are recommended to abate the growing burden of AMR.

## Background

Conventional (“western”) medicine has remained the dominant method of managing various diseases worldwide using therapeutic agents, such as antibiotics (ref). During their circulation, some of these agents remain unused because of various reasons such as reaching their expiry dates, getting contaminated, relief of symptoms, forgetfulness, excessive prescribing by health workers, or side effects (1–3). The World Health Organization (WHO) estimates a daily medical waste of up to 0.5kg per hospital bed, of which 3% is unused medicine globally (4). Furthermore, evidence has shown that up to two-thirds of dispensed medicines in the United States (US) households alone end up unused, leading to a total money wastage of up to 5.4 billion US dollars(6). Such unused medicines if not appropriately disposed end up in the environment and their metabolites have often been found in effluents from sewerage facilities, water bodies, and water for drinking (6–9). It also predisposes to various adverse health threats such as acute poisoning (10), impaired fertility to aquatic life (11), suicide, and accidental death especially in children as well as fueling antibiotic resistance progress-the current global time bomb.In order to minimize those undesired consequences, the United States’ Food and Drug Administration (FDA) formulated guidelines concerning safe disposal of unused drugs. These include: (a) the first and best method is the medicine take-back, a program aimed at increasing safe disposal of unused medicines in order to reduce on the pharmaceutical waste load in communities by returning these unused and /or expired medicines to community pharmacies or drug collection depots (12). (b) Where such take-back programs are unavailable, the unused drug should be mixed with an undesirable substance such as dirt, cat litter, or used coffee grounds. The formed mixture should then be put in a sealable container and thrown into the household’s trash (12). (c) A special case is for controlled medicines such as Morphine, Fentanyl and other Opioids. In the absence of medicine take-back programs, such medicines should be disposed by flushing them in the toilet water system (12). Such medicines are on the FDA’s flush list.

However, despite the existence of these guidelines, majority of the populations worldwide do not adhere to them. Studies done in the US reported that less than 1 % of households disposed via the medicine take back method (13) while more than half of patients disposed their medicine via toilet flushing (14). Similar studies done in India, Bangladesh, China (15–17) and some few African countries (1,18–19) found that dumping into landfills was the commonest method of disposing unused medicine- a very primitive and hazardous method. The exact barriers to safe disposal of unused medicines are not very clear. With the current wide spread easy access to especially over the counter drugs, it is very hard to regulate drug use among populations (20). However, primary prevention of environmental contamination with pharmaceuticals through improvement in the safe drug disposal knowledge and practices is quite an easier and more feasible measure.

In Uganda, very limited data exists on safe drug disposal. A study by Musoke and colleagues (21) found out that majority of homesteads in the general public disposed their unwanted medicine by dumping and burning in rubbish pits- a very dangerous practice. Generally, the few studies that have been done both in Uganda and other Sub-Sahara African countries concerning drug disposal (1, 18–19, 21) have been among the public, and left out the healthcare professional students (HPS) who are at the frontline of prescribing, using, as well as being the primary source of advice regarding rational medicine use to the general public as they dispense these medicines.

This study therefore intended to assess the knowledge, practices, and barriers to safe disposal of unused medicine among undergraduate HPS at Makerere University College of Health Sciences (MaKCHS). Results could guide on establishing appropriate interventions that increase awareness about, as well as mitigating barriers that hinder proper disposal of unused drugs, hence a better health for man, animals, and their shared environment- a step towards achieving the One Health objective.

## Methods

### Study design

Between 1^st^February and 16^th^March 2022, we conducted an online, descriptive, cross-sectional study among undergraduate health professions students of MaKCHS.

### Study area and setting

The study was conducted at MaKCHS. The college is the medical school of Makerere University and is the oldest medical school in Uganda and in East Africa. It is located at the Mulago hill, which is approximately 4 kilometers by road north of Kampala- the country’s capital city. The schools of the college offer undergraduate and postgraduate courses in the biomedical sciences, health sciences, human medicine, and public health, covering a broad range of disciplines and specialties. MaKCHS has about 2,000 undergraduate students pursuing various medical programs.

### Target population

All undergraduate medical students from year one to year five, aged 18 years or older, pursuing any program at MAKCHS which include Bachelor of Medicine and bachelor of Surgery (MBChB), Bachelor of Pharmacy (BPHARM), Bachelor of Nursing (BSN), Bachelor of Cytotechnology (BCYT), Bachelor of Dental surgery (BDS), Bachelor of Medical Radiology (BMR), Bachelor of Environmental Health Science (BEHS), Bachelor of Speech and Language Therapy (BSLT), Bachelor of Biomedical Science (BSB), among others.

### Sample size

The estimated sample size was calculated using the Epi info CDC Sample size calculator software for population survey studies. Using an estimated population size of 2000 students, at a confidence interval of 95%, expected frequency of 50% and a margin of error of 6%, the estimated sample size was 235 participants.

### Study variables

Independent variables included sex, age, program of study, year of study and place of residence. Dependent variables included Knowledge, practices, and barriers regarding safe disposal of unused drugs among HPS.

### Data collection tool

Data was collected using a pre-tested self-administered questionnaire adopted from previous studies done by Maharaj and colleagues (19) and was slightly modified by the investigators using the FDA guide on safe disposal of unused medicines to suit with our setting. It comprised of 4 sections as follows:

**Section 1**: Consisted of7 questions assessing for the participants’ demographic characteristics.

**Section 2**: Consisted of9 questions assessing for participants’ knowledge regarding safe disposal of unused medicine.

**Section 3**: Consisted of 7 questions assessing for participants’ practices regarding safe disposal of unused medicines.

**Section 4**: Consisted of 2 questions assessing for participants’ perceived barriers to safe disposal of unused medicines.

### Data collection procedure

During the time of data collection, though Uganda was out of lock-down, the Standard Operating Procedures to reduce the transmission of COVID-19 disease were still highly emphasized by the Ministry of Health. Therefore, we used WhatsApp Messenger and email for enrolling the study participants. By employing convenience sampling, we identified all the existing WhatsApp groups and personal email addresses of HPS in Makerere University. An Enketo express tool box form link to the questionnaire was sent to the identified eligible participants via WhatsApp groups, personal in-boxing and emailing.

### Quality assurance

The research assistants underwent an intensive training about the research topic, its procedures, techniques, communication skills and data collection. The data collection tool was first tested on ten undergraduate students from the college of Veterinary Medicine, Makerere University and all necessary corrections were made before administering the tool to the final study participants. Submitted data was subjected to data cleaning prior to data analysis to ensure that only complete questionnaires from eligible participants were considered. Entered data was checked properly for accuracy.

### Data analysis plan

The data extracted from fully completed Enketo form questionnaires was entered in Microsoft Excel for cleaning and coding. It was then transferred to STATA (StataCorp) Version 15.1 for analysis and storage. Numerical data was summarized as means and standard deviations. Categorical data was summarized at proportions and frequencies. Blooms cut-off of 80% was used to determine whether a participant has good or poor knowledge and practices concerning safe disposal of unused medicine. Associations between independent and dependent variables were assessed using Chi-square and Fisher’s exact test. A p<0.05 was considered statistically significant.

## Results

A total of 208 responses were received. After data cleaning, 205 responses (response rate = 205/235 = 87.23%) were eligible and included final analysis.

### Demographic characteristics of respondents

Of the 205 respondents, 135(65.9%) were male. The median age was 23 (range: 19–43) years. Most participants (n = 143, 69.8%) were pursuing MBChB. Other demographic characteristics are summarized in Table 1.

### Knowledge about safe disposal of unused medicines

**Figure 1:**
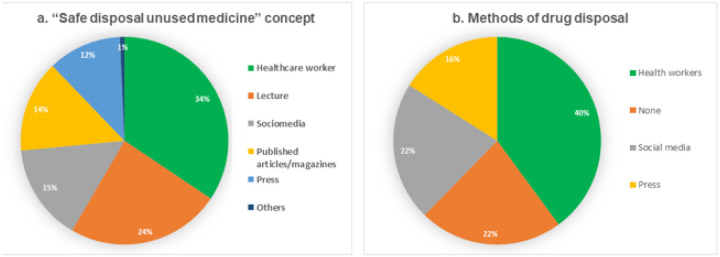
Source of information about safe disposal of unused medicines;a: About the concept, b: about the recommended safe disposal methods

Overall, 157(76.6%) respondents had good knowledge concerning safe disposal of unused medicine. The overall mean knowledge score was 63 ± 18.1 %. However, only a few respondents (n = 131, 36.1 %) knew the recommended approach to dispose unused medicines in absence of the take back program, Table 2. Likewise, participants demonstrated significant level of trust in information distributed by social media and press, Fig. 1.

Descriptively, females, pharmacy students, and students residing in rentals had better knowledge compared to their counterparts, Table 1. However, at logistic regression analysis, only year of study was a significant factor associated with good knowledge about safe disposal of unused medicines, and participants in higher years had more knowledge compared to those in lower years of study, Table 3. There was no statistical difference between age, sex, residence, and study programs.

### Practices

**Figure 2:**
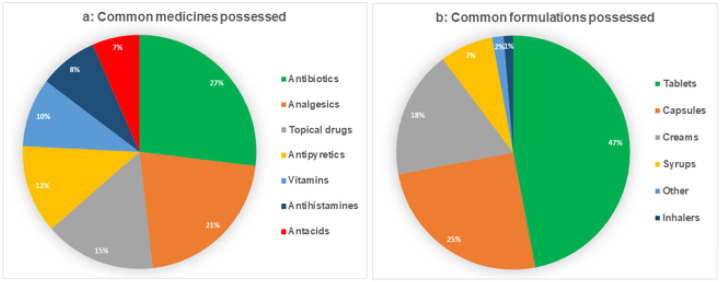
Common drugs and drug formulations possessed at home

Most respondents in this study usually kept unused medicines in their homes and at the time of data collection, about half (n = 102, 49.8%) had some unused medicines at their homes. The most common medicines they kept included antibiotics (27%), analgesia (21 %) and topical drugs (15%) in formulations of tablets (47%), capsules (25%) and creams (18%), Fig. 2.

The major reasons reported for the possession of unused medicines by respondents included keeping them for emergency purposes (67.3%), discontinued after resolved condition (50.2%) and expired drugs (21 %). Participants who disposed unused medicines reported doing so through; household garbage (50.2%), burning with rubbish (28.8%), and flushing them in toilets/sinks (24.9%). Only 18% of participants reported having taken back medicines to pharmacies when they no longer needed them, Table 4.

### Barriers and proposed solutions

**Figure 3:**
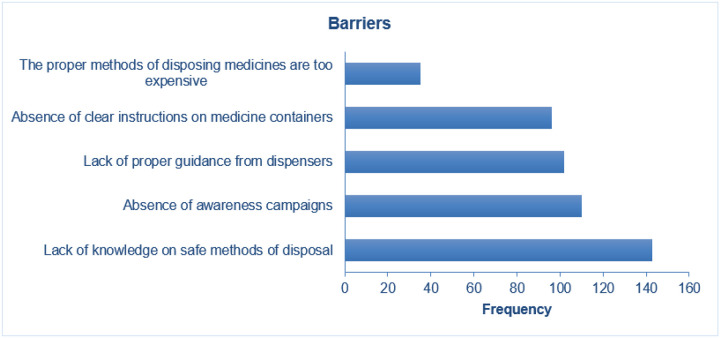
Views a bout the factor hindering practice of safe disposal of unwanted medicine

**Figure 4:**
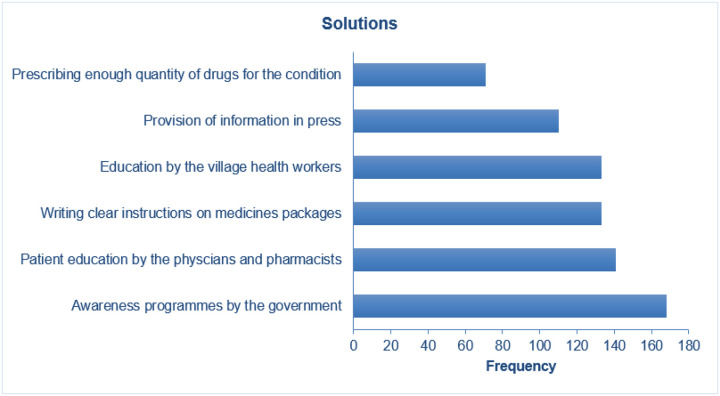
Potential Measures suggested to imSprove practices

The most common reported barriers to practice of safe disposal of unused medicinces included: lack of knowledge, absence of awareness campaigns and lack of proper guidance from dispensers, Fig. 3. Participants recommended that the governement should advocate for awareness campaigns and health workers ensure that patients are well educated and given proper instructions while dispenising drugs, Fig. 4.

## Discussion

In this study, which aimed at investigating the knowledge, practices and barriers to safe disposal of unused medicines among HPS in uganda, we found over three-quarters of respondents had good knowledge about safe disposal of medicine but generally poor practices with regard to this. The main barriers to safe medicine disposal were the inadequate knowledge and insufficient guidance from dispensers.

The good knowledge among most respondents (>76%) is possibly because of some course units in their curricula concerning medicine management, storage, and disposal such as pharmacotherapeutics. This finding correlates with that reported by Nisha and colleagues among dental and medical students in Nepal (22) whose knowledge level about medicine storage and disposal was at about 80% but differs from results by studies in SaudiArabia reported by Bashata and Wajid 2020 among nursing and Pharmacy students(23), among undergraduate and postgraduate pharmacy students (24), as well as among the general public in Ethiopia (25)which found relatively low knowledge levels concerning safe medicine disposal.

Our study also reports that almost half (49.8%) of the respondents had unused medicines in their respective residences and that majority of these were antibiotics. This sets a high risk for antibiotics exposure, access and misuse, which can easily fuel antimicrobial resistance- one of the current leading global health threats. This high posession could possibly be because the category of respondents were HPS with significant knowledge about most of the medicines, hence self prescribe and medicate themselves in case of any illness as earlier studieshave already indicated high levels of self-medication among HPS (26–27).This finding corelates with that by Auta et al among pharmacy students in Nigeria (28). However, this value is quite lower than that reported among many studies done among the general public where posession of unused medicines was above 70% (29–31).This indicates an urgent need for moreantimicrobial stewards among both the HCWs and the general public for a good future of our medicines.

We also report that half of the respondents reported dumping their unused medicines into the household garbage as their main disposal method. This finding is quite low compared to that reported in other countries, where over three-quarters of HPS reported disposing their unused medicines into the household garbage(22–23,28) plus many other studies done among the general public in Ethioopia (25)and indonesia(29) that found a high prevalence of dumping intohousehold garbage.

Surprisingly, less than one-fourth (18%) of respondents reported to have used the take-back method before. This finding is an unfortunate one because this is considered the most safe and recommended method of medicine disposal. This meager value is possibly due to the almost negligibe awareness programmes in the country about safe medicine disposal. It is a very low figure and is congruent with findings among the general public in Kabul(30), Malaysia (32) and India (33) that reported a very low usage of the take back method of disposing medicines. More effort is recommended towards more awareness programmes in order hasten the adoption of theserecommended safe medicine disposal methods by both the HCWs and the general public worldwide.

The major barriers for safe disposal of medicine reported were absence of awareness campaigns and proper guidance from the prescribers. This is not surprising however as negligible efforts have been generally directed towards safe drugs disposal campaigns in Uganda at large. This result is congruent with studies done from other countries (29, 33) where the general public thought that knowledge could be improved by tapping more detailed information and teachings from healthcare workers - the nurses, pharmacists, and doctors.

Only the year of study was associated with knowledge level. This is possibly because students in upper years have had relatively adequate experince with drug management including possible storage and disposal, than those in the lower years of study.This finding is in agreement with that by Nisha et al (22) but albeit different fromother studies that found significant associations between knowledge and a variety of other demographic characters such as gender and program of study (31).

We have a few limitations to our study. Firstly, we used a convenience sampling method, whereby only the students who were willing and ready to participate in the study answered our questions, hence the results may not be generalised. Secondly, the participants answered the questionnnaire via an online self administered tool. Hence, we were not able to confirm some of their actual answers for instance, confirming wether those who said had unused medicines at their residential places actually had them or not. However, the authors ensured that a big response rate was obtained and that all the years of study were relatively balanced at the college, hence these results can be generalised.

## Conclusions

Despite demonstration of a relatively high level of knowledge regarding safe drug disposal, most of the respondents’ practices regarding safe disposal of unused medicines are still suboptimal. Solving the major barriers to good medicine disposal knowledge such as education by dispensers could give amazing results that limit the fueling of unhealthy effects of improper drug disposal.

## Figures and Tables

**Table 1:** Characteristics of participants

Characteristic	Total	Level of Knowledge	
	n (%)	Good, n (%)	Poor, n (%)
**Overall**	N = 205	157(76.6)	48(23.4)
**Age [Median: 23 (range: 19–43)]**		
≤ 23	111 (54.2)	85(76.6)	26(23.4)
> 23	94(45.9)	72(76.6)	22(23.4)
**Sex**		
Male	135(65.9)	101(74.8)	34(25.2)
Female	70(34.2)	56(80)	14(20)
**Religion**		
Roman catholic	82(40)	65(79.3)	17(20.7)
Anglican	64(31.2)	48(75)	16(25)
Pentecost	33(16.1)	23(69.7)	10(30.3)
Others	15(7.3)	13(86.7)	2(13.3)
Muslim	11(5.4)	8(72.7)	3(27.3)
**Study program**		
MBChB	143(69.8)	109(76.2)	34(23.8)
BNS	14(6.8)	10(71.4)	4(28.6)
BPHARM	10(4.9)	12(85.7)	2(14.3)
BSB	8(3.9)	8(80)	2(20)
BDS	16(7.8)	5(62.5)	3(37.5)
Others	14(6.8)	13(81.3)	3(18.8)
**Year of study**		
Year 5	59(28.8)	44(74.6)	15(25.4)
Year 4	44(21.5)	37(84.1)	7(15.9)
Year 3	41(20)	31(75.6)	10(24.4)
Year 2	40(19.5)	34(85)	6(15)
Year 1	21(10.2)	11(52.4)	10(47.6)
**Place of residence**		
Hall	117(57.1)	90(76.9)	27(23.1)
Private hostel	36(17.6)	25(69.4)	11(30.6)
Home	28(13.7)	22(78.6)	6(21.4)
Rental	24(11.7)	20(83.3)	4(16.7)
**Availability of flush toilet at residential place**		
Available	195(95.1)	150(76.9)	45(23.1)
Not available	10(4.9)	7(70)	3(30)

**Table 2:** Participant’s responses on the knowledge questions

Question	Responses	
	correct	Incorrect
Ever heard of “Safe disposal of unused medicines”	160(78)	45(22)
Improper disposal of medicines in the environment can cause undesirable health consequences to human	200(97.6)	5(2.4)
Improper disposal of medicines can reduce the effectiveness of such medicines in treating diseases in the future	129(62.9)	76(37.1)
What do you think is the best way of disposing off unwanted medicines	139(67.8)	66(32.2)
The recommended Home-based way of disposing unwanted controlled medicines e.g. opioids that are in liquid form is	135(65.9)	70(34.1)
Prior to disposing off unwanted medicines in solid form, it is recommended that?	121(59)	84(41)
Where there is no take back programme, solid medicines should be disposed by?	74(36.1)	131(63.9)
What are the recommended routes of disposing unwanted medicines?	161(78.5)	44(21.5)

**Table 3:** Binary logistic regression analysis of the factors associated with good knoweldge of safe disposal of unused medincines

Characteristic	Odds Ratio (95% Confidence Interval)	p-value	Adjusted Odds Ratio (95% Confidence Interval)	p-value
**Age**				
≤ 23	1		1	
> 23	1(0.5,1.9)	0.997	1.1(0.4,2.6)	0.914
**Sex**				
Female	1		1	0.108
Male	0.7(0.4,1.5)	0.407	0.5(0.2,1.2)	
**Religion**				
Anglican	1		1	
Roman Catholic	1.3(0.6,2.8)	0.541	1.5(0.7,3.5)	0.337
Muslim	0.9(0.2,3.8)	0.873	1(0.2,4.4)	0.968
Pentecost	0.8(0.3,1.9)	0.577	0.7(0.3,2.1)	0.553
Others	2.2(0.4,10.7)	0.341	2.1(0.4,11.4)	0.369
**Study program**				
MBChB	1		1	
Others	1.1(0.5,2.2)	0.853	2.1(0.8,5.8)	0.149
**Year of study**				
Year 1	1		1	
Year 2	5.2(1.5,17.4)	0.008	9.7(2.3,40.5)	0.002
Year 3	2.8(0.9,8.6)	0.068	4.2(1.2,14.6)	0.022
Year 4	4.8(1.5,15.6)	0.009	9.5(2.1,43.3)	0.004
Year 5	2.7(0.9,7.5)	0.064	6(1.3,26.6)	0.019
**Place of residence**				
Hall	1		1	
Private hostel	0.7(0.3,1.6)	0.365	0.7(0.3,1.7)	0.370
**Age**				
Rental	1.5(0.5,4.8)	0.492	2(0.5,8.6)	0.368
Home	1.1(0.4,3)	0.852	0.9(0.3,2.9)	0.901
**Availability of flush toilets**				
Available	1		1	
Not available	0.7(0.2,2.8)	0.616	0.7(0.1,4.1)	0.673

**Table 4:** Unused medicine disposal practices among participants

Practices	Frequency	Percentage
**Participants who usually possess some unused or unwanted medicines at their homes**		
Usually have	159	77.6
Usually don’t have	46	22.4
**Participants who possessed any unused medicines at their homes at the time of this survey**		
Do not have	103	50.2
Have at least one drug	102	49.8
**Practiced storage methods for unused medicines**		
Drawer	154	75.1
Suit case	32	15.6
Safety cabinet	4	2
Hand bag	4	2
First aid box	1	0.5
**Factors leading to possession of the unused medicines at home**		
Keeping it for an emergency	138	67.3
Self-discontinuation after the condition resolved	103	50.2
The drugs expired	43	21
Absence of take-back programme	42	20.5
Forgot to take it	35	17.1
The doctor changed the treatment	27	13.2
The doctor prescribed more than needed	26	12.7
Experienced adverse effects to the medicine	17	8.3
I do not know the right way / place to dispose them off	15	7.3
**Practiced disposal methods for unused medicine**		
Throwing them away into household garbage	103	50.2
By burning them at a rubbish pit	59	28.8
By flushing them in the toilet/ sink	51	24.9
By taking them back to the pharmacy or hospital	37	18
By throwing them into the incinerator	32	15.6
By selling or giving them to other people	25	12.2
By throwing or dumping the at landfills	14	6.8

## Data Availability

The datasets used and/or analyzed during the current study are available from the corresponding author on reasonable request.

## Abbreviations

COVID-19: Corona virus disease-2019
HEPI: Health Professional Education Partnership Initiative
HPS: Health Profession Students
MAKCHS: Makerere University, College of Health Sciences
MBChB: Bachelor of Medicine and Bachelor of Surgery
MHREC: Mulago Hospital Research and Ethics Committee
